# Imaging of carotid artery dissection

**DOI:** 10.3402/jchimp.v2i2.18645

**Published:** 2012-07-16

**Authors:** Mansoor Mozayan, Carlton Sexton

**Affiliations:** 1 Chief Resident, Department of Medicine, Medstar Union Memorial Hospital, Baltimore, MD, USA; 2 Department of Radiology, Medstar Union Memorial Hospital and University of Maryland School of Medicine, Baltimore, MD, USA

A 32-year-old woman presented to the emergency room complaining of a headache and blurred vision in her left eye. Two weeks before presentation she felt ‘sinus pressure’ and heard a swishing sound in her left ear associated with pain and tightness on the left side of her head, face, and neck, most prominent at the angle of the jaw. She sought emergent attention when she felt a ‘gritty sensation’ in her left eye and noted that her left eyelid was red and droopy. On evaluation vitals were normal. Her forehead showed no vasomotor or sudomotor changes. She had an injected left conjunctiva with miotic pupil (left 3 mm, right 5 mm) which was reactive to light and accommodation. Corneal fluorescein uptake and fundoscopic exam were normal. Vision was 20/20 in both eyes. Extraocular movements were normal, but the patient complained of pain in the left eye. The higher cortical, other cranial nerve, motor, sensory, and cerebellar functions were normal. Imaging studies showed a left internal carotid artery dissection extending up to the base of the skull.

Craniocervical artery dissection presents in a variety of ways including headache and neck pain, oculomotor Horner's syndrome as in our patient, and signs of cerebral ischemia that may be delayed by hours or days. Though rare (2.5 to 3 cases per 100,000 in the general population) (1, 2), the diagnosis is made because of awareness of its clinical manifestations and because of advances in non-invasive imaging, chiefly color duplex ultrasonography, CT angiography, MRI and MR angiography, and conventional catheter angiography. These rely on imaging of the mural pathology, principally an intramural hematoma, sometimes resulting in and sometimes resulting from an intimal tear.

Color duplex ultrasound, not performed in our patient, detects mural hematoma and thrombus as a thickened hypoechoic wall. Because most internal carotid artery dissections begin near the carotid bulb in the mid-neck where ultrasound has easier access to the vessel, sensitivity ranges from 71% without symptoms of ischemia to 95% when there is clinical cerebral ischemia (3, 4).

**Fig. 1:**
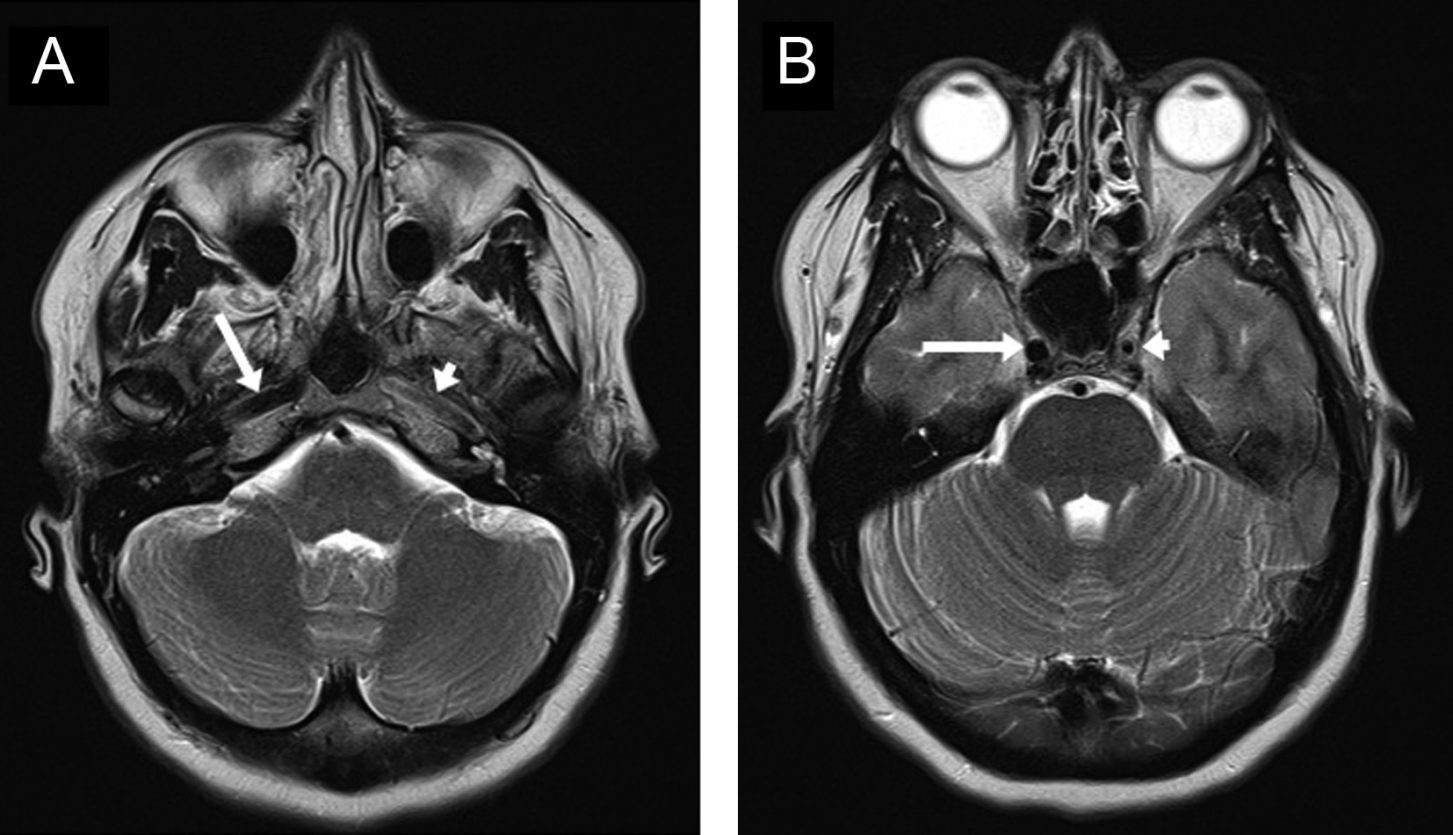
Axial T2-weighted MRI demonstrating dissection of internal carotid artery. (A) The petrous left internal carotid artery (short arrow) has diminished flow, demonstrated by a loss of black signal, compared to the normal right internal carotid artery (long arrow). (B) The cavernous left internal carotid artery (short arrow) with a smaller lumen compared to the normal right internal carotid artery (long arrow).

**Fig. 2:**
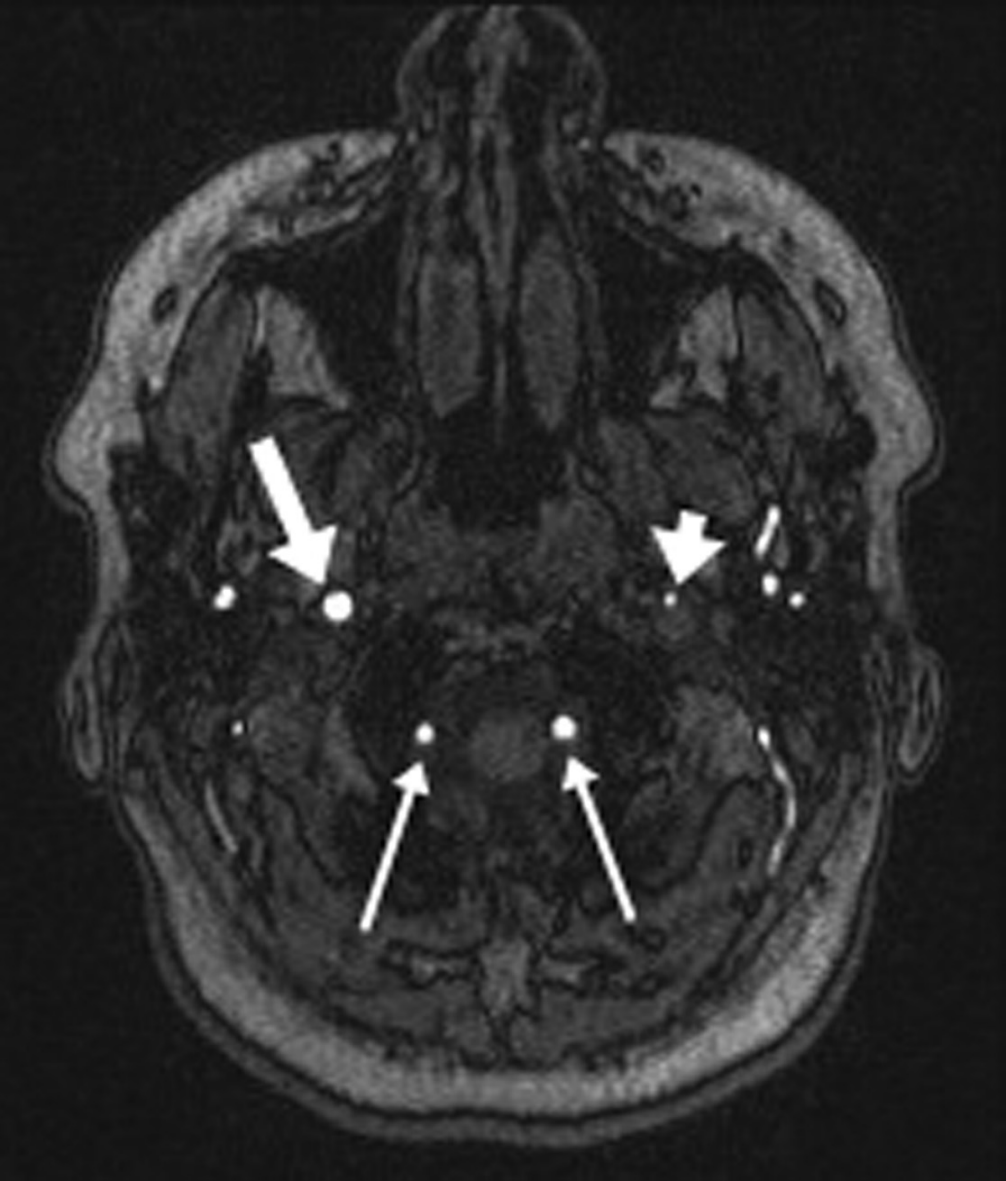
2D MR angiography demonstrating the severely narrowed lumen shown by the small focus of flow-related signal of the left internal carotid artery (short arrow) is compared to the normal right internal carotid artery (long arrow) and to the vertebral arteries (thin arrows).

In evaluation of Horner's syndrome, where the autonomic nerves are compressed as they course along with the enlarged, dissected petrous carotid artery in the skull base, MRI and MR angiography of the head and neck will both make the diagnosis and evaluate the brain supplied by the affected vessel (Figs 1 and 2). Sensitivity and specificity of MR imaging is reported as 84 and 99%, respectively, when the criteria of expanded external diameter and narrowed lumen are applied (5).

**Fig.3:**
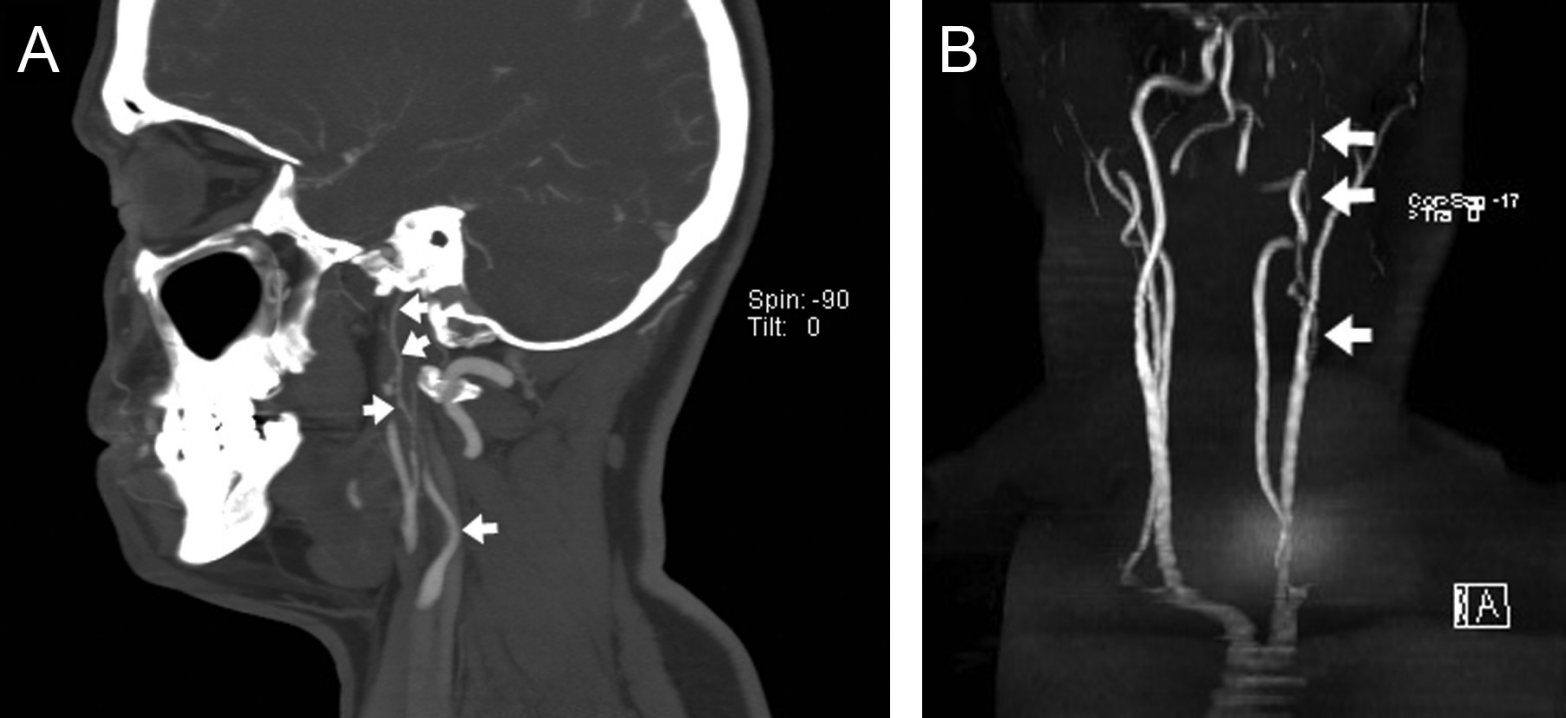
The long segment of stenosis, but not occlusion, of the left internal carotid artery (short arrows) is more definitively demonstrated on CT angiography (A) on a sagittal maximum intensity projection than on MR angiography (B) on coronal maximum intensity projection.

CT angiography provides better spatial resolution than MR imaging to evaluate the caliber of a severely narrowed vessel (Fig. 3), although visualization of soft tissue structures close to bone, like the petrous carotid artery, is compromised by beam hardening artifact. Internal carotid artery dissection often begins beyond the vessel origin, unlike most atherosclerotic narrowing, and in cross-section on the axial images is represented classically by an eccentric contrast-filled lumen with thickened crescentic vessel wall. Enlargement of the overall vessel diameter results in autonomic nerve compression in the non-distensible petrous carotid canal, resulting in Horner's syndrome as in our patient. Contrast-outlined intimal dissection and dissecting aneurysm are other manifestations of the disease, but were not present in our patient.

Our patient was anticoagualated and her symptoms completely resolved. Treatment with anticoagulation therapy is usually sufficient, and dissections usually go on to heal. Decisions about reducing or terminating anticoagulation can be made using follow-up MRI/MRA or CTA. Surgical or endovascular treatment is rarely required. Recovery depends on the severity of the vascular compromise, with a mortality rate of up to 5%. Among patients who suffer headaches, a 90% symptom resolution rate is observed within 1 week (6).
